# Toward a More Sustainable Paper Industry: The Contribution of the Hydrothermal Carbonization for Solid Residues

**DOI:** 10.1021/acsomega.5c11140

**Published:** 2026-02-12

**Authors:** Luca Taglieri, Alberto Gallifuoco, Katia Gallucci, Luciano Fratocchi

**Affiliations:** Department of Industrial and Information Engineering & Economics, 9303 University of L’Aquila, Piazzale Ernesto Pontieri 1, L’Aquila 67100, Monteluco di Roio, Italy

## Abstract

The paper industry has historically relied on virgin raw materials as the primary resource for production. Moreover, it requires high energy and water consumption and use of chemicals like chlorine. In response to these environmental challenges, the paper industry is progressively moving toward more sustainable practices aligned with circular economy’s principles. However, the shift to recycling introduces new challenges, particularly the generation of new solid typologies of residues, such as pulper rejects and fine screen debris. This study investigates hydrothermal carbonization (HTC) as a circular solution for transforming such two solid residue streams into an effective solid fuel. More specifically, it verifies the technical feasibility of such technology on a laboratory scale. Moreover, the energy potential and environmental impacts of the obtained fuel are evaluated. Finally, it is checked whether the obtained hydrochar is compliant with the EU directive regarding Solid Recovered Fuel (SRF). Our findings confirm that HTC is an effective solution for the transformation of investigated solid residues. Moreover, all obtained hydrochars have a higher level of high heating value when compared with the original inputs (up to 83,6%). In this respect, the operating severity (250 °C) and liquid-to-solid ratio emerged as primary levers to raise the heating value while curbing halogens, mercury, and SRF-critical inorganics. Finally, the obtained hydrochars meet EU legislation in terms of energy and environmental performance, reaching even SRF Class 1 levels. This in turn confirms that HTC process parameters and feedstock mix can be properly tuned to deliver SRF-grade solids with stable, high-quality combustion behavior.

## Graphical abstract

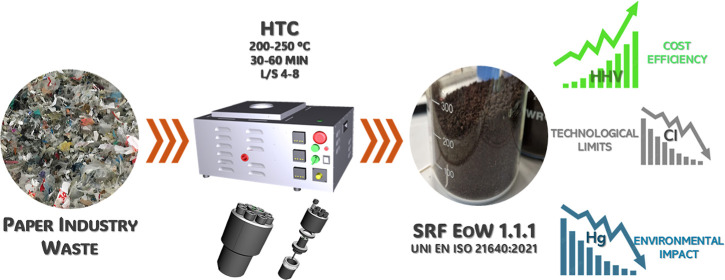

## 1 Introduction

The paper industry has historically relied on virgin raw materials as the primary resource for production (e.g., wood fibers). This dependency has significant environmental impacts, including deforestation, loss of biodiversity, and the degradation of ecosystems.^1^

Moreover, the pulping process involves chemical and mechanical treatments that are highly energy- and water-intensive.^2–4^ At the same time, the bleaching of pulp to produce high-quality paper involves chemicals like chlorine, which can generate toxic effluents harmful to aquatic ecosystems.^5^

In response to these environmental challenges, the paper industry is progressively moving toward more sustainable practices aligned with circular economy principles.^6,7^ This transition includes improving wastewater treatment and minimizing resource consumption, especially reducing dependence on virgin raw materials. In this respect, it is worth noting that at a global level, about 60% of paper production already relies on recycled fibers (more than 50% in Europe).^8^

The growing use of these secondary raw materials (SRMs), particularly from municipal solid waste (MSW), has become central to this shift, with an increasing focus on packaging applications (e.g., cardboard) due to the rise in the demand for sustainable materials. However, such a shift to recycling introduces new challenges, particularly the generation of new solid typologies of residues (different from traditional sludge), such as pulper rejects (Ps) and fine screen debris (Ss).

P is generated during the recycling process when wastepaper is broken down. It constitutes approximately 75% of the solid waste that comes from the paper recycling process. This waste is an unavoidable byproduct due to the collection and sorting of recyclable paper, which inherently contains impurities and noncellulosic materials that are inseparably bound to the paper. It consists of a mixture of materials not recyclable in the paper manufacturing process, including plastics, metal items or fragments, glass, sand, certain nonpulpable paper types, and cellulose fibers adhered to these materials.^9^ The Best Available Techniques (BAT) Reference Document for the paper sector outlines the components of pulper rejects, highlighting their variability depending on long-term changes in consumer habits.^10^

Ss are the result of the fine screening process, that is, the separation stage within a paper mill where the pulp is passed through fine screens or sieves to remove unwanted particles, such as small debris, impurities, or foreign materials. These residues may include small unwanted paper particles, plastic, or other impurities that need to be eliminated from the production process to obtain high-quality pulp.^11^ These materials, although unavoidable in the paper recycling process, represent a critical hurdle in achieving a circular and sustainable paper production chain.

Currently, Ps and Ss are either incinerated or sent to a landfill. Both approaches present significant drawbacks: on one side, the incineration is characterized by high economic costs (since residues are classified as a waste, which implies collection and treatment services’ costs for paper manufacturers), emissions, and limited energy recovery due to the high moisture content and low calorific value of such a material.^12^ On the other side, the landfilling is even more unacceptable due to stringent environmental regulations and growing disposal costs.^7^ However, Ps and Ss incineration is highly critical not only because of its low efficiency but also for operational problems due to its heterogeneous composition (such as, slagging, increased ash production, and the need for more complex flue gas cleaning systems).^13^

Therefore, innovative solutions are requested for effectively managing Ps and Ss.^14^ In this respect, the conversion of paper industry residues into valuable fuel often requires pretreatment technologies to enhance their energy performance. Several methods have been explored in recent years to address this challenge, including thermal and mechanical processes. Each technology offers distinct advantages and limitations depending on the feedstock characteristics, operational conditions, and end-use requirements. Recent studies highlight the potential of hydrothermal processes for treating heterogeneous,^15^ multimaterial residues.^16–19^ The technology mimics the natural coalification process, accelerating it from geological time scales to a few hours.^20^

Within the paper industry, research on hydrothermal carbonization (HTC) has so far been largely confined to the treatment of sludge,^21–27^ while, to the best of our knowledge, its application to solid production residues such as P and S has not yet been systematically explored.

Against this background, the following two primary research questions (RQs) are addressed in this study:

RQ 1 Can HTC be effectively applied to paper industry solid residues, such as Ps and Ss?

RQ 2 What are the energy potential and environmental impact of the obtained hydrochar?

Moreover, no previous research has yet addressed the potential of this HTC to support an End-of-Waste (EoW) pathway for such residues, enabling their valorization within a circular economy framework. Therefore, two further research questions emerge:

RQ 3 Could the obtained hydrochar be classified as Solid Recovered Fuel (SRF) compliant with EU directives?

To shed new light on such RQs, HTC was applied to Ps and Ss obtained in the wastepaper recycling process at the Burgo Group’s Avezzano plant.

The findings, developed at the laboratory scale, demonstrate the effectiveness of HTC as a circular economy solution for paper industry residues. By converting these materials into a valuable solid fuel, HTC can significantly reduce disposal costs, improve resource recovery, and support the industry’s transition toward sustainable energy practices within the limitations of the present bench-scale study and the simplified industrial scenario considered.

## 2 Theoretical Framework

### 2.1 Traditional Technologies for Ps and Ss Pretreatment

Several methods have been explored to identify effective pretreatments requested for improving Ps and Ss energy performances; among them, thermal and mechanical processes were proposed.

As regards thermal processes, two main alternatives are generally adopted: thermal drying and torrefaction. Thermal drying is a widely adopted pretreatment method aimed at reducing the moisture content of wet residues. By exposure of the material to controlled heat, typically through direct or indirect dryers, the water content can be significantly lowered, thereby increasing the lower heating value (LHV) of the fuel. Two main benefits characterize such a technology. First of all, an improved combustion efficiency, as less energy is wasted in vaporizing moisture, and storage and handling properties of the dried material are enhanced. However, thermal drying is often energy-intensive and may offset the overall energy recovery benefits, especially when applied to high-moisture residues, such as pulper rejects.

On the contrary, torrefaction is a mild pyrolysis process, carried out at temperatures ranging from 200 to 300 °C in an oxygen-depleted environment. This process produces a carbon-rich, hydrophobic solid product with enhanced fuel properties, including (a) increased calorific value, (b) improved grindability, and (c) reduced biological activity, making the material more stable for long-term storage. While torrefaction effectively improves fuel quality, it requires additional energy inputs and may release volatile organic compounds (VOCs), which necessitate gas-cleaning systems for environmental compliance.^28^

Mechanical processes are represented by mechanical densification, such as pelletization and briquetting.^29^ They are widely used to compress low-density residues into high-energy-density solid fuels.^30^ These processes improve the physical properties of the material, including (a) increased bulk density and (b) uniform particle size, which enhances combustion efficiency.^31^

However, for residues such as Ps and Ss, mechanical densification encounters significant limitations. Due to their heterogeneous nature and elastic properties, the compressed product tends to regain its initial volume after pressurization. This phenomenon drastically reduces the efficiency of the densification process, limiting its applicability and resulting in fuels with inconsistent properties in terms of bulk density, moisture content, and calorific value. Such variability hinders both the predictability of performance in combustion systems and compliance with the quality standards typically required for SRF. Additionally, the high moisture content of these residues exacerbates the issue, hindering the compaction process and further lowering the calorific value of the resulting product. Given these challenges, mechanical densification alone is not a viable solution for upgrading paper industry residues.

### 2.2 HTC

Conventional thermal and mechanical pretreatments, previously considered, are limited by the high moisture, heterogeneity, and elastic behavior of paper industry residues. Hydrothermal treatment has been proposed as a potential alternative, since it can process wet feedstocks under moderate temperatures (180–250 °C)^32^ in a pressurized water environment, where hydrothermal reactions may stabilize the material and improve its energy properties.^33^ HTC can efficiently process heterogeneous and high-moisture materials,^34^ offering multiple benefits:^35^ enhanced calorific value^36^ and dewaterability^37^ and lower environmental impact.^38^ The more recent extant literature offers several studies claiming that hydrothermal processes are the proper recipe for treating multimaterial residues providing multiple advantages.^39,40^ Recent findings indicate that HTC process water itself can be a valuable source of platform chemicals, further improving the sustainability of the technology.^41,42^

Additionally, HTC should have a minimal impact on water consumption compared to the large use of such a resource in a water-intensive paper industry. Finally, when comparing available technologies, HTC emerges as the most effective approach, offering significant enhancements in energy properties for waste streams.^43^

## 3 Methodology

### 3.1 The Organization Contest

To solve earlier RQs, a research methodology based on a single case study was adopted, given the exploratory nature of the contribution. More specifically, a purposive sampling approach was implemented since a case study selected through predefined criteria would increase the study’s reliability.^44^ The HTC application to the pretreatment of Ps and Ss was experimented in the Burgo Group’s paper mill located in Avezzano (Central Italy). The plant produces containerboard from 100% recycled paper. The plant generates approximately 15,000 tons of solid residues annually, composed primarily of Ps and Ss. The management of such waste represents a significant economic and environmental burden, with disposal costs estimated at €200 per ton. The incineration of residues is the method currently employed by Burgo for waste management.

The Burgo plant in Avezzano (Italy) furnished the two endogenous solid residues.

### 3.2 Experimental Procedure

Given the research aims described above, an experimental campaign was conducted under controlled laboratory conditions. The HTC process was applied to pulper rejects and fine screen debris collected from the Burgo Group’s paper mill. The experiments were designed to explore the influence of temperature (200 and 250 °C), residence time (30 and 60 min), and water-to-solid mass ratio (4 and 8) on the quality of the produced hydrochar.

For each set of operating conditions, HTC experiment was carried out once for each set of operating conditions as happens in HTC extant literature.^45–48^

HTC experiments were performed in a bench-scale batch reactor based on the design previously reported by Di Giacomo et al.^49^ The reactor was made of AISI 316 stainless steel and had an internal volume of about 200 mL. It was housed inside an electrically heated oven equipped with band heaters (1250 W each) and a PID temperature control. Thermocouples located inside the reactor (immersed in the reacting slurry) and in the heating system were connected to a dedicated HTC controller, which maintains the process temperature at the desired set-point. The internal reactor temperature was measured by a K-type (Chromel/Alumel) mineral-insulated thermocouple with an insulated hot junction, housed in an Inconel 600 sheath (1.6 mm OD, 150 mm length), and connected to the controller for a precision of ±2%.

During the isothermal holding time, the reactor was magnetically stirred to enhance heat and mass transfer and to avoid the formation of cold zones. In addition, the temperature of the heating bands was constrained to be at most 90 °C higher than the reactor set-point, thereby limiting wall–fluid temperature differences and preventing local overheating at the reactor wall. At the end of each run, the reactor was rapidly cooled by immersion in a water bath to quench the reaction.

All samples were ground to a particle size of approximately 5 mm and then dried in an oven at 105 °C for 24 h. They were subsequently sealed in bags and stored until further use. Table 1 lists the names of the samples along with the moisture content and chemical composition.

**Table 1:** Feedstocks Used for HTC Tests

type of waste	pulper reject	fine screen debris
name of the sample	P	S
water content [% wt]	25.7	43.5
C [% wt_dry_]	50.40	45.49
H [% wt_dry_]	8.89	6.41
HHV [MJ/kg]	24.06	18.65
Cl [% wt_dry_]	0.2244	0.07554
Hg [% wt_dry_]	0.00010	0.00001
Sb [mg/kg]	3	3
As [mg/kg]	1	1
Cd [mg/kg]	2	1
Cr [mg/kg]	21	17
Co [mg/kg]	10	6
Mn [mg/kg]	15	19
Ni [mg/kg]	37	30
Pb [mg/kg]	5	6
Cu [mg/kg]	38	15
Ti [mg/kg]	1	1
V [mg/kg]	5	6

As the reactor was closed, the pressure was monitored as autogenous pressure along with the pressure created by the produced gases. A 1:4 or 1:8 ratio of dry sample to deionized water was used to load the reactor. The hydrochars were obtained at reaction temperatures of 200 and 250 °C. The heating process of the reactor was as follows: (a) the contents were heated at a rate of approximately 8 °C/min until reaching the set reaction temperature, (b) once the target temperature was reached, the reactor was kept at that temperature for 30 and 60 min, and (c) after the residence time, the reactor was cooled to room temperature by immersing it in a water bath, which typically took around 1 min. The produced gases were released in a fume hood, and the liquid was separated from the solid hydrochar through vacuum filtration. The hydrochar was dried in an oven at 105 °C for 24 h and then stored in a vial for further analysis.

Obtained hydrochar samples were labeled through a code containing the following information: starting feedstock (“P”, “S”, or “M” when the mix of 75% of P and 25% of S was utilized); process temperature (200 or 250 °C); residence time (30 or 60 min); and liquid-to-solid ratio (4 or 8). As an example, “P_250_30_8” means a sample of pulper reject that has undergone a hydrothermal process at 250 °C with a residence time of 30 min and a water/solid mass ratio equal to 8.

#### 3.2.1 Characterization of Hydrochars’ Quality

In order to evaluate the quality of the obtained hydrochars, we performed the following parameters:

(a) Carbon and hydrogen content of the feedstocks in input and obtained hydrochar were performed with the standard method^52^ using a PerkinElmer 2400 Series II.
(b) Higher heating value (HHV) and the LHV calculated on the dry solids’ content of all solid samples obtained using a drying oven set to 105 °C in accordance with the guidelines of international standard.^50^ More specifically, HHV and LHV parameters were calculated according to Channiwala and Parikh,^51^ which developed a general correlation for predicting the calorific values of solid, liquid, and gaseous fuels from their elemental composition. This method has been widely adopted due to its accuracy and reliability in estimating HHV and LHV.^53–55^

(c) Inorganic elements and heavy metals (namely, Cl, Hg, Sb, As, Cd, Cr, Co, Mn, Ni, Pb, Cu, Tl, and V) were determined as follows. Dried hydrochar samples were ground and homogenized, and approximately 0.5 g of each sample was subjected to acid digestion (HNO_3_/HCl) according to a standard procedure. The resulting solutions were analyzed by inductively coupled plasma–optical emission spectrometry (ICP–OES) to quantify both major inorganic constituents and trace metals, using appropriate blanks and calibration standards for quality control. In particular, the set of inorganic elements and heavy metals analyzed corresponds to those required by UNI EN ISO 21640:2021 for SRF classification. The analytical determinations needed to evaluate the quality of obtained hydrochars through the described parameters were assessed performing three measures for each sample. Data reported in the paper represent the mean values; the corresponding standard deviations were always lower than 5% of the mean, a level which is considered acceptable in previous HTC studies.^56–58^

## Classification as SRF EoW

A central aspect of this work is linked to the second research question, namely, whether the hydrochar obtained from HTC treatment can be classified as a SRF that meets the EoW criteria established by national and European regulations. The successful classification of hydrochar would facilitate its valorization as an alternative fuel, reducing the disposal costs associated with paper industry residues, while contributing to the transition toward a circular economy.

In order to effectively classify the materials studied as SRF and facilitate efficient trading, as well as promote safe utilization in energy conversion and gain public trust, the present study references the UNI-EN-ISO 20640:2021 standard.^59^

In Europe, the management of waste, including the transformation into SRF is governed by the Waste Directive (2008/98/EC). This directive establishes principles for waste treatment, promoting prevention, recycling, and the conversion of waste into resources, while aiming to protect the environment and human health. The directive also defines criteria for when waste ceases to be waste and becomes a resource, which is essential for SRF, as it enables energy recovery from waste.

Specifically, in Italy, the legislative framework for waste management and energy recovery includes Legislative Decree No. 205/2010 and the Environmental Code (Legislative Decree No. 152/2006).

These regulations set out specific criteria to produce SRF, including calorific value, chemical composition, and environmental standards that SRF must meet for use as fuel. Importantly, the compliance assessment involves not only energy parameters but also the concentration of heavy metals, and in particular, if the concentration of these elements exceeds the threshold values set by the standard, the material cannot be classified as SRF, regardless of its calorific performance.

The SRF classification system, as presented in Table 2, is determined through assessing specific limit values for three crucial fuel characteristics. These characteristics are divided into five class levels, and the SRF must be assigned a class number from 1 to 5 for each one of them. In the table, only the classes with a gray background represent “SRF EoW”. The assumed moisture content for the solids is 30% for the wastes as received and 20% for the hydrochars, considering the best dehydratability.^37^

**Table 2:** SRF Classification System

characteristic	classes				
	1	2	3	4	5
LHV [MJ/kg (ar^a^)]	≥25	≥20	≥15	≥10	≥3
Cl [%wt_dry_]	≤0.2	≤0.6	≤1.0	≤1.5	≤3
Hg [mg/MJ(ar)]	≤0.02	≤0.03	≤0.05	≤0.1	≤0.15

maximum admitted inorganic content	
Sb [mg/kg]	≤50
As [mg/kg]	≤5
Cd [mg/kg]	≤4
Cr [mg/kg]	≤100
Co [mg/kg]	≤18
Mn [mg/kg]	≤250
Ni [mg/kg]	≤30
Pb [mg/kg]	≤240
Cu [mg/kg]	≤500
Ti [mg/kg]	≤5
V [mg/kg]	≤10

a ar = as received.

## 4 Results and Discussion

### 4.1 HTC Technical Effectiveness

Adopting the previously described methodology, 24 hydrochar samples were obtained, eight for each feedstock (namely, P and S) and for their mix. Figure 1 shows the visual appearance of the two inputs (P and S) and the hydrochar obtained through their mix (M_250_60_8).

**Figure 1:**
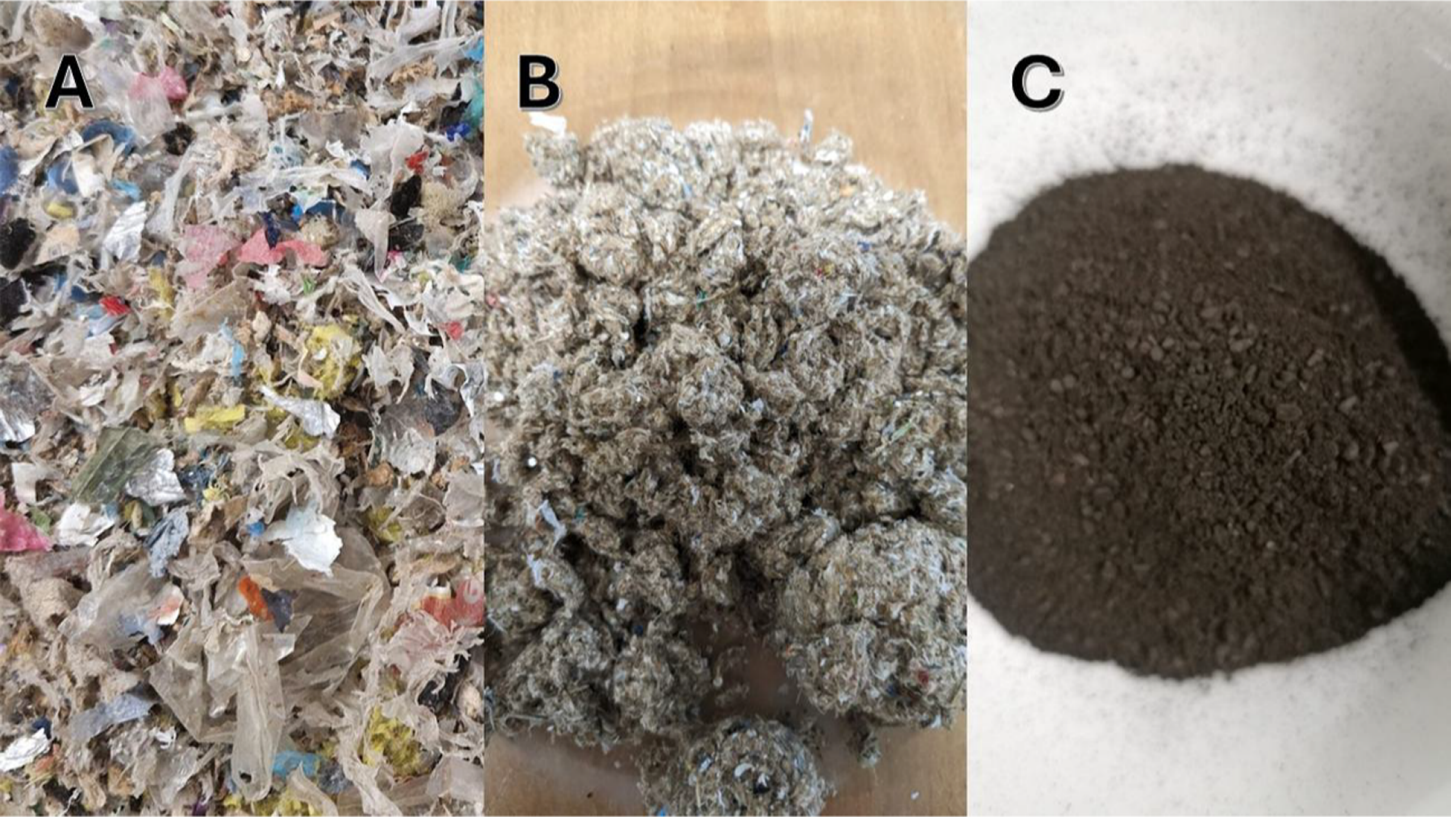
Visual appearance of pulper reject (A), fine screen debris (B), and hydrochar M_250_60_8 (C).

As earlier described, the quality of obtained hydrochars was primarily evaluated through C, H, and HHV levels (Table 3).

**Table 3:** List of Solid-Phase Properties for Assessing HTC Performances

name of the sample	C [% wt_dry_]	H [%wt_dry_]	HHV [MJ/kg]
P_200_30_4	66.50	9.47	34.36
P_200_60_4	54.13	8.26	25.69
P_250_30_4	69.45	10.05	36.49
P_250_60_4	61.79	9.15	31.09
S_200_30_4	48.18	6.93	21.44
S_200_60_4	54.99	8.04	26.27
S_250_30_4	61.64	4.70	24.01
S_250_60_4	58.31	6.37	25.14
P_200_30_8	74.27	10.57	37.32
P_200_60_8	59.85	9.74	30.35
P_250_30_8	76.36	14.66	44.02
P_250_60_8	78.01	13.65	44.17
S_200_30_8	48.62	6.06	19.71
S_200_60_8	47.37	5.77	20.79
S_250_30_8	63.08	7.15	27.65
S_250_60_8	68.37	6.31	29.07
M_200_30_4	51.16	7.24	24.07
M_200_60_4	53.66	8.28	24.75
M_250_30_4	72.11	11.89	39.31
M_250_60_4	71.21	12.61	38.37
M_200_30_8	50.58	6.84	21.72
M_200_60_8	56.17	8.87	26.84
M_250_30_8	65.44	10.12	32.77
M_250_60_8	70.17	9.20	34.22

The obtained values of the C content and HHV clearly show higher values when compared to the two initial parameters of adopted feedstocks (P and S). Therefore, HTC emerges as an effective pretreatment for such type of solid residues.

#### 4.1.1 Hydrochars’ Energy Performances

When considering (RQ2), Table 3 summarizes energy performances of each of the obtained hydrochars, which must be compared with the ones characterizing the initial solid residues, 24.06 MJ kg^–1^ (P) and 18.65 MJ kg^–1^ (S), respectively. All obtained hydrochars have a higher level of HHV, reaching a maximum of 44.17 MJ kg^–1^ for P_250_60_8 (+83,6%) and 29.07 MJ kg^–1^ for S_250_60_8 (+55.9%). Carbon enrichment followed the same trend, peaking at 78.01 wt % for P_250_60_8. Across all hydrochar samples, HHV and carbon content were strongly correlated, confirming that the HTC severity primarily governs energy densification via carbonization and deoxygenation pathways.^60^ A special note deserves the use of a mixed feed (M): the baseline blended HHV (22.71 MJ kg^–1^) was upgraded to 39.31 MJ kg^–1^ in M_250_30_4 (+73.1%) and to 34.22 MJ kg^–1^ in M_250_60_8 (+50.7%). These outcomes indicate that mixing streams do not dilute performance; instead, it can deliver hydrochars with balanced fuel properties. This evidence becomes more relevant when considering that such mixing is quite attractive from an industrial standpoint, since it allows use simultaneously of both solid residues.

The trends in the elemental composition reported in Table 3 are consistent with these energy performances. Increasing HTC temperature from 200 to 250 °C systematically enhanced carbon enrichment in all residues. The average C content increased from about 63.7 to 71.4 wt % for P, from 49.8 to 62.9 wt % for S, and from 52.9 to 69.7 wt % for M, while the corresponding HHV gains were from 31.93 to 38.94 MJ kg^–1^ for P, from 22.05 to 26.47 MJ kg^–1^ for S, and from 24.35 to 36.17 MJ kg^–1^ for M. Hydrogen followed a similar trend for P and M (from 9.5 to 11.9 wt % and from 7.8 to 11.0 wt %, respectively), whereas for S it remained essentially in the range of 6–7 wt %, indicating that temperature mainly drives deoxygenation and carbon densification rather than hydrogen enrichment in this residue. At fixed temperature, residence time and liquid-to-solid ratio exerted a more nuanced, feedstock-specific influence on C, H, and HHV. For instance, for P at 250 °C and LSR = 4, extending residence time from 30 to 60 min (P_250_30_4 → P_250_60_4) decreases both C (from 69.45 to 61.79 wt %) and HHV (from 36.49 to 31.09 MJ kg^–1^), whereas for S longer residence times slightly increase both parameters. Moreover, for P at 250 °C and 60 min, increasing the liquid-to-solid ratio from 4 to 8 (P_250_60_4 → P_250_60_8) markedly enhances C (from 61.79 to 78.01 wt %) and H (from 9.15 to 13.65 wt %), in line with the strong HHV increase from 31.09 to 44.17 MJ kg^–1^, thus confirming the role of water load in promoting solvolysis of oxygenated species and energy densification.

Further useful findings emerge when analyzing the impact of the process severity (*T* and residence time) and liquid-to-solid ratio (LSR) on the hydrochars’ HHV levels. When considering *T*, moving from 200 to 250 °C increased mean HHV from 31.93 to 38.94 MJ kg^–1^ (P), 22.05 to 26.47 MJ kg^–1^ (S), and 24.35 to 36.17 MJ kg^–1^ (M). At the same time, residence time effects were sample-dependent (e.g., P_250_30_4 → P_250_60_4 decreases from 36.49 to 31.09 MJ kg^–1^, whereas S_250_30_8 → S_250_60_8 increases from 27.65 to 29.07 MJ kg^–1^), suggesting competing condensation/devolatilization vs secondary cracking/fragmentation depending on matrix composition. Overall, temperature is the dominant driver of energy densification, while residence time exerts a second-order, feedstock-specific influence.

The LSR effect was also feedstock-specific. For P at 250 °C and 30 min, increasing LSR from 4 to 8 raised HHV from 36.49 to 44.02 MJ kg^–1^ (+7.53 MJ kg^–1^), indicating enhanced solvolysis/partitioning of oxygenated species at a higher water load. For S, LSR changes yielded smaller HHV shifts (e.g., S_250_60_4 = 25.14 to S_250_60_8 = 29.07 MJ kg^–1^). For M, the highest HHV was observed at LSR equal to 4 (M_250_30_4), pointing to a process “sweet spot” where adequate water is present for hydrolysis without excessive solubilization of energy-bearing intermediates. These results support tailoring LSR by residue type to balance hydrolysis extent and solid-phase carbon retention.

### 4.2 Hydrochars’ Environmental Impacts

A further parameter adopted for evaluating the hydrochars’ quality regards the concentration of inorganic elements in the hydrochar samples (Table 4). Such a parameter, obtained following the procedure described in the previous section, is also critical for assessing the environmental quality of the obtained fuels (RQ2). More specifically, for each sample, it was possible to verify if the obtained hydrochar is compliant with the levels requested to be classified as an SRF. Eleven out of 24 samples resulted out of the ranges, mainly due to the amount of Ni.

**Table 4:** List of Solid-Phase Inorganic Content [mg/kg_dry_]­^a^

name of the sample	Cl	Hg	Sb	As	Cd	Cr	Co	Mn	Ni	Pb	Cu	Tl	V
P_200_30_4	46.45	0.008	3	1	2	21	10	15	37^a^	5	38	1	5
P_200_60_4	54.01	0.009	3	1	1	17	6	19	30^a^	6	15	1	6
P_250_30_4	108.5	0.01	16	1	1	75	3	5	71^a^	16	472	0	3
P_250_60_4	21.05	0.005	3	2	1	61	3	25	55^a^	17	242	1	6
S_200_30_4	5.93	0.006	26	1	1	107^a^	3	5	93^a^	26	661^a^	1	2
S_200_60_4	7.329	0.008	3	2	1	51	58^a^	28	181^a^	19	175	0	6
S_250_30_4	6.664	0.003	3	1	0	9	3	4	12	5	20	1	5
S_250_60_4	10.77	0.003	3	2	1	50	4	19	47^a^	16	33	1	14^a^
P_200_30_8	6.112	0.01	3	1	1	15	3	4	13	5	22	1	7
P_200_60_8	4.367	0.005	9	2	1	72	5	36	86^a^	37	76	1	12^a^
P_250_30_8	10.51	0.003	3	1	1	54	3	1	14	2	19	1	1
P_250_60_8	6.487	0.005	3	0	1	10	3	1	17	2	29	1	1
S_200_30_8	3.396	0.004	13	1	1	17	3	1	34^a^	7	53	0	1
S_200_60_8	3.927	0.006	3	1	0	18	3	1	23	2	40	1	1
S_250_30_8	5.084	0.004	3	0	1	9	3	3	10	3	12	1	3
S_250_60_8	5.562	0.006	3	1	0	9	3	3	10	3	14	1	2
M_200_30_4	8.863	0.004	3	2	1	19	3	2	15	10	69	1	5
M_200_60_4	18.81	0.002	3	2	1	21	3	4	19	10	38	1	4
M_250_30_4	67.88	0.003	3	1	1	16	3	5	25	4	31	1	4
M_250_60_4	12.93	0.005	6	1	0	15	3	1	29	3	34	1	4
M_200_30_8	5.879	0.005	10	1	1	81	3	1	76^a^	19	448	0	12^a^
M_200_60_8	12.85	0.01	3	1	1	22	3	1	46^a^	5	49	1	6
M_250_30_8	12.57	0.003	11	1	1	15	3	1	23	3	41	0	1
M_250_60_8	60.27	0.005	3	1	0	10	3	1	23	2	25	1	5

a Values exceed UNI-EN-ISO 20640:2021 thresholds.

For instance, S_200_30_4 shows elevated As and Ni, while M_200_30_8 presents high Cr and Ni, typical of lower-severity HTC in which metals remain in the solid matrix. The behavior of Ni and Cr at increasing HTC temperatures can be interpreted in terms of the HTC reaction mechanism. As the severity increases, the organic matrix of the papermaking residues is progressively decomposed by hydrolysis, dehydration, and decarboxylation reactions, which lowers the solid yield and transfers a large fraction of C, H, and O to the liquid and gaseous phases. In contrast, Ni and Cr are mainly present in inorganic, nonvolatile, and poorly soluble forms, so that their absolute mass is largely conserved in the solid phase. As a consequence, their contents expressed on a dry-mass basis increase in the hydrochars produced at higher temperatures, even though no additional contamination is introduced. Moreover, the formation of a more condensed carbon matrix and new mineral phases at higher HTC temperatures favors the immobilization of Ni and Cr in more stable forms, which is consistent with the generally low leaching potential expected for these metals in the resulting SRF. When considering the process parameters, it emerges that increasing temperature generally reduced apparent metal retention in the hydrochar (e.g., M-series at 250 °C vs 200 °C), consistent with redistribution into the aqueous phase and/or structural changes that limit solid-phase association.^61^ Therefore, in order to ensure a friendly overall environmental performance, the HTC process must operate at sufficiently high severity (high temperature and long residence times) and appropriately manage aqueous effluents within the limitation that only the solid phase was analyzed.

Given these promising results, further experimental activities should be implemented on a pilot-scale reactor in order to verify the technical and economic feasibility of the scalability of the HTC process. Moreover, potential environmental impacts should be carefully evaluated. In this respect, water and energy consumption deserve specific attention. As far as the use of water concerns, a first-pass scale-up for a 15,000 t y^–1^ line operated under M_250_60_8-type conditions indicates an additional process water demand of ∼100,000 t y^–1^, that is just around 4% of the mill’s current total water use. Therefore, the HTC process at an industrial level is compatible with integration into existing water circuits and with potential for internal recirculation after adequate treatment. Future investigation should devote specific attention to the possibility of activating the process of water recirculation in order to further reduce the HTC environmental impact.

When considering energy consumption, it is worth noting that the optimized hydrochar (LHV in the mid-30 MJ kg^–1^ range) could offset roughly 11% of annual fossil-fuel consumption at the site, delivering both operating-cost savings and GHG reductions while complying with SRF/EoW criteria. It should be stressed that this industrial-scale assessment is intended as a preliminary, order-of-magnitude scenario based on a simplified scale-up of the bench-scale results and on a set of explicit assumptions on plant integration and the SRF outlet. Finally, future research should also investigate the impact of off-gas emissions.

### 4.3 SRF Classification and EoW Eligibility

In order to investigate RQ3, it must be verified that each obtained hydrochar meets SRF parameters and, at the same time, respects the levels of LHV, Cl, and Hg shown in Table 2. Obtained data are summarized in Figure 2 (LHV), Figures 3 (Cl) and 4 (Hg).

**Figure 2:**
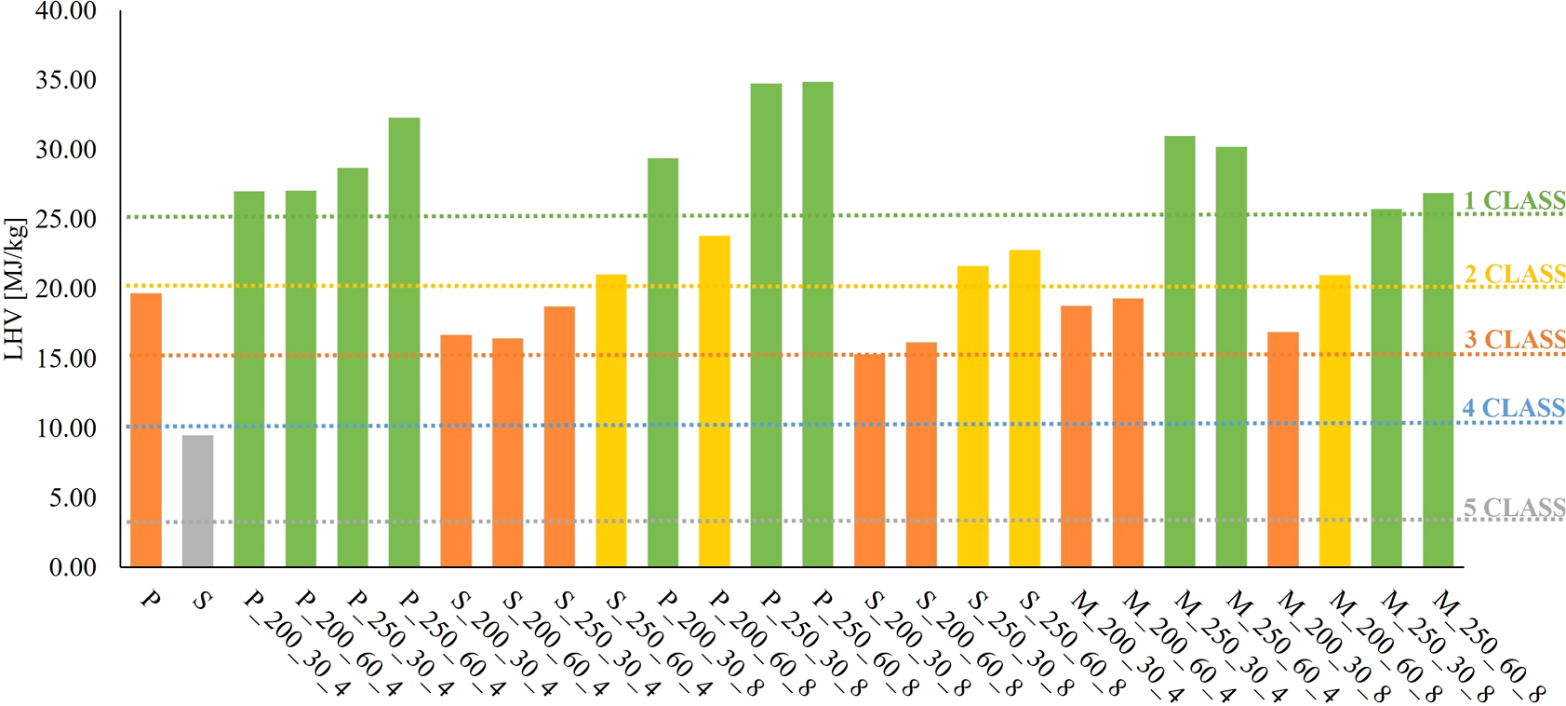
Results of SRF classification in terms of LHV.

**Figure 3:**
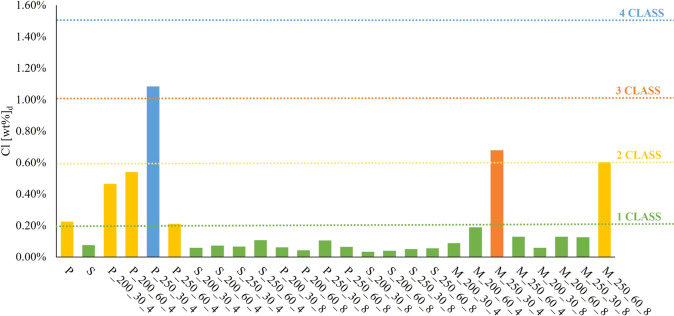
Results of SRF classification in terms of chlorine content.

**Figure 4:**
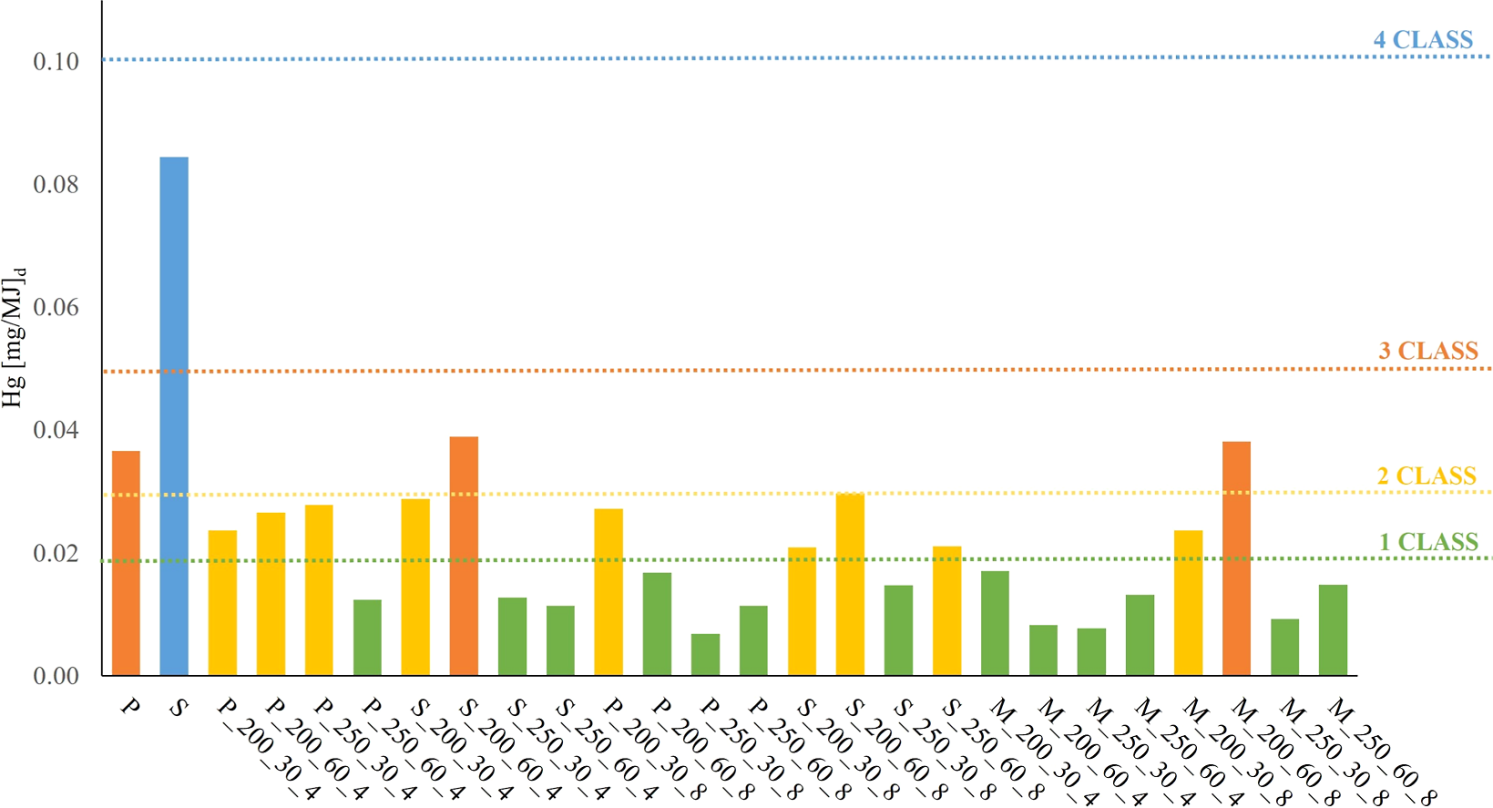
Results of SRF classification in terms of mercury content.

Considering the preference of industrial operators for hydrochars obtained from a mixed feed (M), M_250_30_8 and M_250_60_8 emerge as the most promising samples since they combine high-energy content with low Cl and Hg content. These low levels of potentially harmful elements make them environmentally favorable for industrial applications, as they meet stringent regulatory standards while minimizing the risk of releasing pollutants during combustion. Finally, it is worth noting that the two hydrochars under discussion are characterized by high levels of severity (mainly in terms of *T*); therefore, our results confirm that HTC process parameters and feedstock mix can be properly tuned to deliver SRF-grade solids with stable, high-quality combustion behavior. The ability to use mixed residue streams without compromising fuel quality opens new avenues for scaling up the HTC process in an industrial setting.

## 5 Conclusions

Bench-scale HTC was adopted to convert two paper-mill residue streams into energy-dense hydrochars with properties compatible with SRF classes relevant to EoW pathways. Overall, the present study demonstrates the technical feasibility of using HTC to convert papermaking solid residues into SRF-compatible fuel at the laboratory (bench) scale.

The operating severity (250 °C) and liquid-to-solid ratio emerged as primary levers to raise LHV (as-received) while curbing halogens, mercury, and SRF-critical inorganics. Under selected conditions, several hydrochars—most notably from a mixed feed—mapped to EoW-compliant SRF bands, indicated a pragmatic valorization route for multimaterial residues otherwise destined to disposal. A first-pass scale-up suggests that additional water demand is manageable within typical mill circuits and that the resulting SRF can offset a nontrivial portion of site fossil heat requirements. The cost-benefit considerations reported herein (e.g., potential fossil-fuel offset and operating-cost implications) should be regarded as preliminary, order-of-magnitude estimates derived from bench-scale results under idealized assumptions on process integration and SRF outlet; a complete techno-economic assessment with sensitivity analysis will be addressed in future work.

Future work will prioritize pilot-scale validation with continuous operation to derisk scale-up (heat integration, solids’ handling, and closed-loop water management), alongside systematic feedstock mapping to quantify how variability propagates to SRF class and emissions. Process optimization will include moisture management for direct LHV­(ar) tuning, selective removal of SRF-critical elements, and treatment and reuse strategies for the aqueous phase to minimize freshwater intake. Collectively, these steps will consolidate the industrial readiness of HTC as a circular EoW-aligned solution for the paper industry.
